# Evaluation of Maternal Inflammatory Biomarkers in Preterm Prelabor Rupture of Membranes: A Systematic Review and Meta-Analysis

**DOI:** 10.3390/medicina62010020

**Published:** 2025-12-22

**Authors:** Sandra Ioana Neamțu, Mihai Sava, Alina Simona Bereanu, Raluca Maria Bădilă, Ioana Roxana Codru, Bogdan Ioan Vintilă, Simina Mustățea, Oana Stoia, Radu Chicea

**Affiliations:** 1 Department of Intensive Care, County Emergency Clinical Hospital, Bld. Corneliu Coposu 2-4, 550245 Sibiu, Romania; 2 Faculty of Medicine, Lucian Blaga University of Sibiu, Lucian Blaga Street 2A, 550169 Sibiu, Romania; 3 Department of Pneumology, County Clinical Hospital, Filozofilor Street 3-5, 550196 Sibiu, Romania; 4 Department of Cardiology, County Emergency Clinical Hospital, Bld. Corneliu Coposu 2-4, 550245 Sibiu, Romania; 5 Department of Obstetrics and Gynecology, County Emergency Clinical Hospital, Bld. Corneliu Coposu 2-4, 550245 Sibiu, Romania

**Keywords:** preterm prelabor rupture of membranes, interleukin-6, maternal inflammatory biomarkers, C-reactive protein

## Abstract

*Background and Objectives:* Preterm prelabor rupture of membranes (PPROM) is a significant obstetric complication associated with increased maternal and neonatal morbidity and mortality. Inflammation plays a central role in its pathophysiology, and maternal inflammatory biomarkers have gained increasing attention as potential predictors of disease onset and adverse outcomes. *Materials and Methods:* This systematic review and meta-analysis synthesized evidence from PubMed, Scopus and Web of Science databases evaluating maternal inflammatory biomarkers—particularly interleukin-6 (IL-6)—in women with PPROM compared with controls. Eligible studies assessed biomarker levels in serum, plasma, or amniotic fluid and reported quantitative outcomes. Data were pooled using random-effects models, and heterogeneity was quantified using the *I*^2^ statistic. *Results:* A total of 23 studies involving 2841 participants were included. Maternal IL-6 concentrations were significantly elevated in PPROM compared with controls in both maternal serum (pooled SMD = 1.72; 95% CI: 1.15–2.29; *p* < 0.001) and amniotic fluid (SMD = 2.84; 95% CI: 2.01–3.67; *p* < 0.001). CRP showed a moderate association (SMD = 0.98; 95% CI: 0.61–1.36; *p* < 0.001), whereas IL-8 and TNF-α displayed inconsistent relationships. *Conclusions:* Elevated maternal IL-6 concentrations, particularly in amniotic fluid, are strongly associated with PPROM and adverse perinatal outcomes. IL-6 demonstrated superior diagnostic and prognostic value compared with other inflammatory markers. These findings support IL-6 as a promising biomarker for early risk identification and individualized the management of high-risk pregnancies.

## 1. Introduction

Preterm prelabor rupture of membranes (PPROM) represents a major obstetric challenge, accounting for approximately one-third of all preterm births and contributing substantially to global neonatal morbidity and mortality [1]. It is defined as the rupture of fetal membranes before 37 weeks of gestation and prior to the onset of labor. The etiology of PPROM is multifactorial, involving mechanical, biochemical, and inflammatory mechanisms that compromise amniotic membrane integrity [2]. Among these mechanisms, inflammation is increasingly recognized as a central driver of membrane rupture, intrauterine infection, and subsequent preterm birth [3,4,5].

Proinflammatory cytokines, chemokines, and matrix metalloproteinases disrupt the extracellular matrix and promote uterine contractility [6]. Interleukin-6 (IL-6), a multifunctional cytokine produced by decidual cells, macrophages, and fetal membranes, plays a pivotal role in mediating these inflammatory cascades [7,8]. Elevated IL-6 levels in maternal serum and amniotic fluid are strongly associated with microbial invasion of the amniotic cavity, histologic chorioamnionitis, and adverse neonatal outcomes, including early-onset sepsis and bronchopulmonary dysplasia [9,10,11,12,13].

Several studies have shown that IL-6 may outperform conventional markers such as C-reactive protein (CRP) and white blood cell count in predicting infection and preterm delivery. Others, however, report inconsistent results, likely due to methodological heterogeneity and varying gestational contexts [14,15]. Beyond IL-6, other maternal biomarkers—including interleukin-8 (IL-8), tumor necrosis factor-alpha (TNF-α), and procalcitonin—have been examined, but their diagnostic reliability remains uncertain [16,17].

Given these discrepancies in the literature, this systematic review and meta-analysis evaluate maternal inflammatory biomarkers in PPROM, focusing primarily on IL-6. The study quantifies the association between IL-6 levels and PPROM, compares its diagnostic and prognostic performance with other biomarkers, and identifies research gaps that could inform future clinical applications [18]. This systematic review and meta-analysis aims to provide quantitative evidence on maternal IL-6 as a predictive biomarker for PPROM and associated adverse outcomes.

Despite the increasing number of studies evaluating inflammatory biomarkers in PPROM, existing evidence remains fragmented, and previous reviews have not provided a unified quantitative assessment focused specifically on maternal IL-6 across serum and amniotic fluid matrices. The novelty of the present systematic review and meta-analysis lies in its comprehensive synthesis of maternal inflammatory biomarkers, with an emphasis on IL-6 as the most widely studied marker, while comparing its diagnostic and prognostic performance with other cytokines.

## 2. Material and Methods

### 2.1. Study Design and Registration

This systematic review and meta-analysis were conducted in accordance with the Preferred Reporting Items for Systematic Reviews and Meta-Analyses (PRISMA) 2020 statement [19]. All methods followed a predefined protocol developed to ensure transparency and reproducibility; however, the protocol was not prospectively registered in PROSPERO or any other registry. Full compliance with PRISMA is documented, and the completed PRISMA 2020 checklist is provided in the Supplementary Materials.

### 2.2. Literature Search Strategy

A comprehensive literature search was performed in PubMed/MEDLINE, Scopus, and Web of Science (WoS) databases from inception to May 2025. The following search terms and Boolean operators were used: “preterm prelabor rupture of membranes” OR “preterm premature rupture of membranes” OR “PPROM” AND “interleukin-6” OR “IL-6” OR “inflammatory marker” OR “biomarker” AND “maternal” OR “serum” OR “amniotic” OR “plasma”. Detailed search strategies for PubMed/MEDLINE, Scopus, and Web of Science databases are provided in Table 1. Both MeSH (Medical Subject Headings) terms in PubMed and Emtree terms in Scopus were used, combined with free-text keywords.

The search strategy was adapted for each database to account for indexing differences. Search filters were not restricted by language or publication year. Reference lists of relevant reviews and included articles were manually screened to identify additional eligible studies.

### 2.3. Eligibility Criteria

The eligibility criteria were defined according to the PICO framework.

- Population (P): Pregnant women diagnosed with PPROM before 37 weeks of gestation were included. Control groups consisted of women with intact membranes, either at term or preterm, with no clinical or histologic evidence of infection.
- Intervention/Exposure (I): The primary exposure of interest was the maternal inflammatory levels, including IL-6, C-reactive protein (CRP), interleukin-8 (IL-8), tumor necrosis factor-alpha (TNF-α), and related cytokines, measured in serum, plasma, or amniotic fluid.
- Comparison (C): The comparator group included women without PPROM or intra-amniotic infection, matched by gestational age where available.
- Outcomes (O): The main outcomes were biomarker concentrations and their association with clinical endpoints, including chorioamnionitis, microbial invasion of the amniotic cavity, neonatal sepsis, and gestational age at delivery.
- Study design: Eligible studies included observational (case–control and cohort) and interventional studies that reported quantitative biomarker data (mean ± SD or median with IQR). Case reports, reviews, conference abstracts, and animal studies were excluded.

#### Selection of Studies for Meta-Analysis

Among the 23 studies included in the qualitative synthesis, only those providing extractable quantitative data were eligible for inclusion in the meta-analysis. Studies were included if they reported maternal IL-6, CRP, IL-8, or TNF-α concentrations in serum or amniotic fluid, provided a control group with intact membranes, and presented data in a form convertible to means and standard deviations. Studies were excluded from the quantitative synthesis if they lacked numerical biomarker values, reported only *p*-values, did not include a comparator group, used biological matrices that could not be analytically combined, or represented overlapping patient cohorts.

Using these criteria, 15 studies contributed data to the serum IL-6 meta-analysis and 8 studies contributed to the amniotic-fluid IL-6 meta-analysis, while fewer studies reported comparable data for CRP, IL-8, or TNF-α. Only studies with moderate or high methodological quality (Newcastle–Ottawa Scale ≥ 6) were included in the quantitative pooling to ensure analytic robustness.

### 2.4. Data Extraction

Two independent reviewers screened all titles and abstracts, assessed full-text eligibility, and extracted data using a standardized form. Extracted information included author and year of publication, country and study design, sample size, gestational age at sampling, biomarkers evaluated and assay methods, diagnostic criteria for PPROM, and maternal and neonatal outcomes (chorioamnionitis, neonatal sepsis, gestational age at delivery). Disagreements were resolved through discussion and consensus.

### 2.5. Quality Assessment

The methodological quality of included studies was evaluated using the Newcastle–Ottawa Scale (NOS) for observational studies [13]. According to this scale, studies scoring ≥7 points were considered high quality, 5–6 moderate quality, and ≤4 low quality.

### 2.6. Statistical Analysis

The primary meta-analysis was performed using a random-effects model with restricted maximum likelihood (REML) estimation of between-study variance and Hartung–Knapp adjustment for confidence intervals, which provides improved control of type I error in the presence of heterogeneity. Effect sizes were summarized as standardized mean differences (SMD) with 95% confidence intervals (CI). In addition, we report the 95% prediction interval, representing the expected range of effects in future comparable studies. Heterogeneity was quantified using the *I*^2^ statistic (25%, 50%, and 75% indicating low, moderate, and high heterogeneity). Publication bias was assessed using funnel plots and Egger’s regression test.

Sensitivity analyses included:

(i) Leave-one-out influence diagnostics;
(ii) Alternative estimators of τ^2^ (Paule–Mandel and DerSimonian–Laird);
(iii) Trim-and-fill assessment for small-study effects;
(iv) Robust variance estimation when multiple effect sizes originated from a single study.

All primary analyses were performed using Stata 17 (StataCorp, College Station, TX, USA) with the meta, metan, metabias, and metainf routines. RevMan 5.4 (Cochrane Collaboration, Oxford, UK) was used only for supplementary visualization (alternative forest plots) and to verify that results were consistent across platforms. All meta-analytic estimates were synthesized using published summary statistics, as individual participant-level data were not available. When required, reported medians and interquartile ranges were converted to means and standard deviations using established methods.

## 3. Results

### 3.1. Study Selection

**Figure 1:**
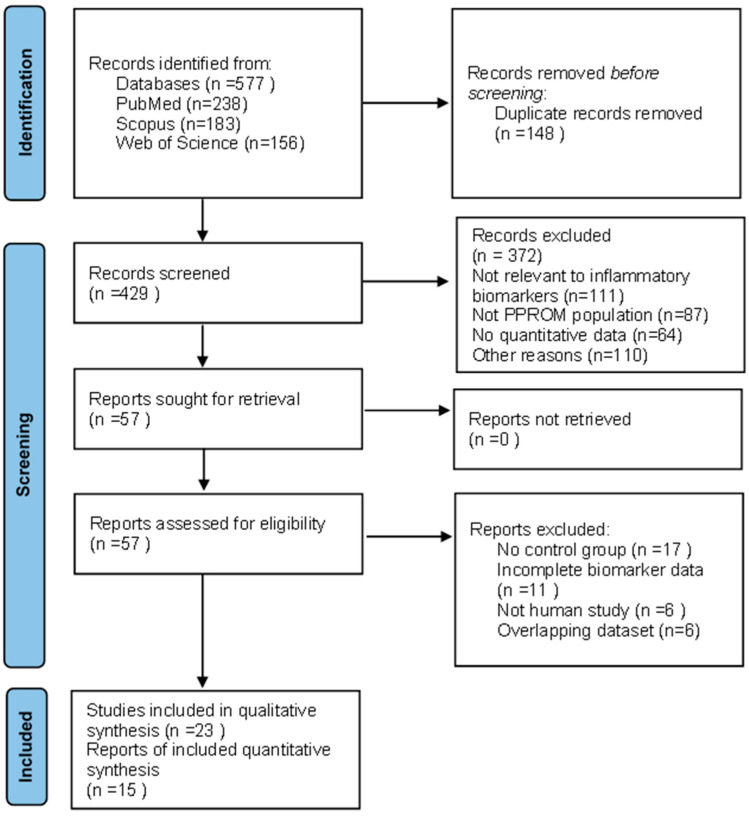
PRISMA 2020 flow diagram summarizing study selection and exclusion steps at each stage (identification, screening, and inclusion).

The database search identified 577 records (PubMed n = 238; Scopus n = 183; Web of Science n = 156). After removing 148 duplicates, 429 unique articles were screened by title and abstract, of which 372 were excluded (111 not relevant, 87 not PPROM, 64 without quantitative biomarker data, 110 other reasons). Fifty-seven full-text articles were assessed for eligibility, and 34 were excluded. A total of 23 studies were included in the qualitative synthesis, and 15 were eligible for meta-analysis. The study selection process is summarized in Figure 1.

### 3.2. Characteristics of Included Studies

The 23 included studies (published 2000–2024) involved 2841 participants (1387 PPROM; 1454 controls). Most were prospective cohort studies. IL-6 was the most frequently assessed biomarker; several studies also evaluated CRP, IL-8, or TNF-α. Biomarkers were measured using ELISA, chemiluminescent assays, or multiplex bead-based platforms. Study characteristics are summarized in Table 2.

#### Quality Assessment

Study quality was evaluated using the Newcastle–Ottawa Scale (NOS). Scores ranged from 6 to 9, indicating moderate to high quality across studies. A detailed summary of NOS scores for each included study is presented in Table 3.

### 3.3. Maternal Serum IL-6 Levels

**Figure 2:**
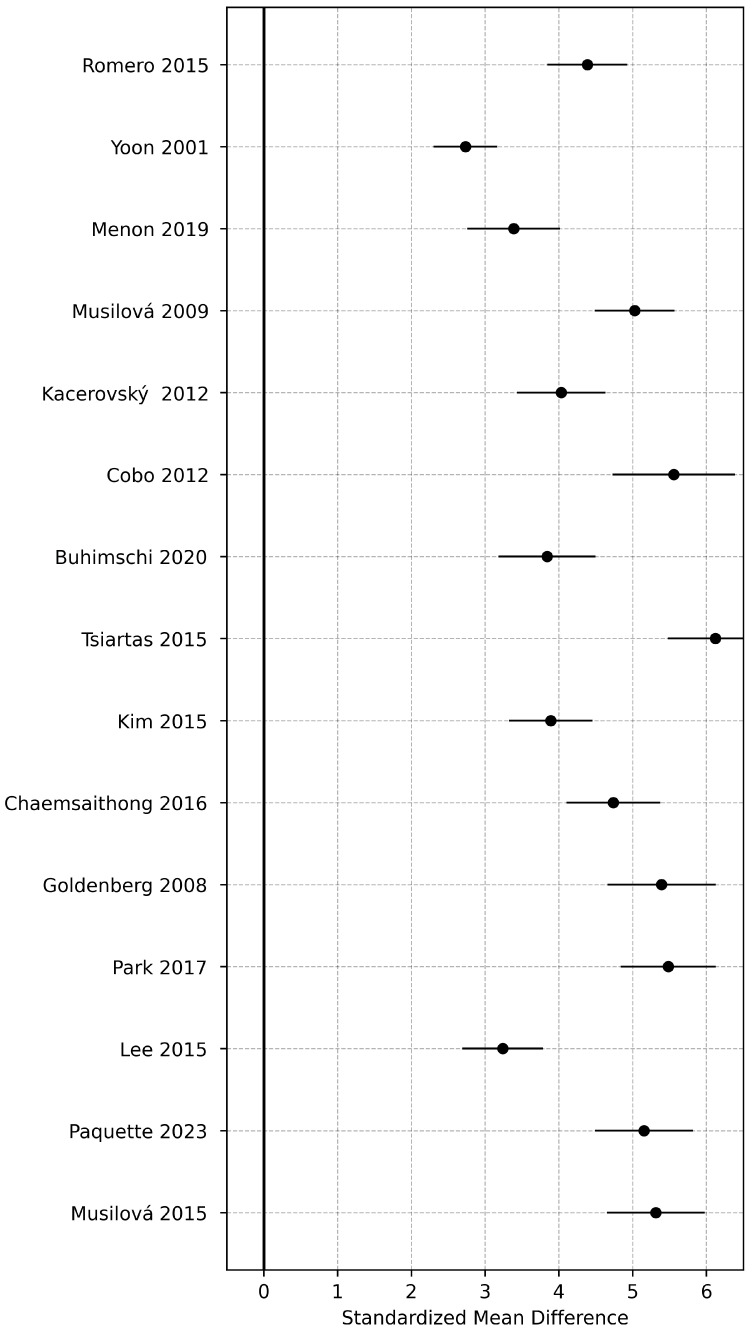
Forest plot of standardized mean differences (SMD) for maternal serum IL-6 concentrations comparing PPROM and control groups [1,2,3,4,7,8,12,13,15,17,18,20,21,22,25].

Fifteen studies evaluated maternal serum IL-6 concentrations. The pooled analysis showed significantly higher IL-6 levels among women with PPROM compared with controls (pooled SMD = 1.72; 95% CI: 1.15–2.29; *p* < 0.001). Heterogeneity was moderate (*I*^2^ = 68%), and sensitivity analyses excluding individual studies did not materially alter the pooled estimate (range, SMD = 1.61–1.79). The forest plot summarizing these results is shown in Figure 2.

### 3.4. Amniotic Fluid IL-6 Levels

Eight studies reported amniotic fluid IL-6 concentrations. Pooled analyses revealed markedly elevated levels among PPROM cases compared to controls (SMD = 2.84; 95% CI: 2.01–3.67; *p* < 0.001). Heterogeneity was substantial (*I*^2^ = 79%), reflecting differences in sampling time and assay methodology. Sensitivity analyses confirmed the robustness of the association.

### 3.5. Other Inflammatory Biomarkers

Meta-analysis of secondary biomarkers showed significantly elevated maternal CRP levels in PPROM (pooled SMD = 0.98; 95% CI: 0.61–1.36; *p* < 0.001). In contrast, IL-8 and TNF-α demonstrated smaller and statistically inconsistent associations. Due to insufficient data, pooled analyses were not feasible for procalcitonin and white blood cell count.

### 3.6. Heterogeneity and Publication Bias

Across biomarkers, heterogeneity ranged from moderate to high (*I*^2^ = 68–79%). Subgroup analyses by region showed slightly higher pooled estimates in Asian cohorts. Egger’s regression test was performed for the serum IL-6 meta-analysis (15 studies) and showed no evidence of publication bias (*p* = 0.21). As fewer than ten studies were available for other biomarkers, Egger’s test was not conducted for those analyses.

### 3.7. Summary of Findings

Maternal IL-6 levels were consistently elevated in PPROM across biological matrices, with larger effect sizes observed in amniotic fluid. CRP showed moderate association, whereas IL-8 and TNF-α demonstrated weaker and inconsistent findings. A summary of pooled effects is presented in Table 4.

Sensitivity analyses using REML with Hartung–Knapp adjustment yielded similar pooled effects and prediction intervals, and trim-and-fill analyses indicated minimal small-study effects.

## 4. Discussion

### 4.1. Summary of Evidence

This systematic review and meta-analysis provide comprehensive evidence that maternal inflammatory biomarkers, particularly IL-6, are strongly associated with PPROM. Across 23 studies and 2841 participants, both serum and amniotic-fluid IL-6 concentrations were markedly elevated in women with PPROM compared with controls. Serum IL-6 showed a large pooled effect (SMD ≈ 1.7), while amniotic-fluid IL-6 demonstrated an even greater effect size, supporting its role as a sensitive indicator of intra-amniotic inflammation [1,3,4,28,29].

Other biomarkers, including C-reactive protein (CRP), IL-8, and tumor necrosis factor-alpha (TNF-α), demonstrated weaker and less consistent associations. CRP showed moderate elevation among PPROM cases, whereas IL-8 and TNF-α yielded smaller and statistically inconsistent findings [11,14,24,30]. These results confirm that IL-6 remains the most robust and reproducible biomarker across studies.

Across biomarkers and study designs, heterogeneity was moderate to high (*I*^2^ ≈ 65–79%), with variations attributable to sampling time, assay methodology, and differences in diagnostic definitions. Nonetheless, sensitivity analyses affirmed the stability of the IL-6 estimates.

### 4.2. Interpretation of Findings

IL-6 is biologically plausible as a key mediator of PPROM pathophysiology. Produced by decidual cells, macrophages, and fetal membranes, IL-6 responds rapidly to microbial invasion or sterile inflammation. It activates prostaglandin synthesis, stimulates matrix metalloproteinases, and contributes to extracellular matrix degradation—mechanisms implicated in membrane weakening and rupture [5,6,22,23]. The higher effect size in amniotic fluid reflects its proximity to the site of inflammation and its established diagnostic utility for microbial invasion of the amniotic cavity and histologic chorioamnionitis [9,10,17,20].

In contrast, CRP and leukocyte-based markers reflect later or systemic inflammatory responses and therefore lack the sensitivity observed with IL-6. IL-8 and TNF-α may be influenced by maternal comorbidities, differing assay sensitivities, and variable thresholds across studies, explaining their inconsistent performance.

Beyond diagnostic applications, IL-6 may have prognostic relevance. Elevated maternal and amniotic-fluid IL-6 levels have been linked to early-onset neonatal sepsis, bronchopulmonary dysplasia, and other inflammation-driven morbidities [1,2,4]. Integration of IL-6 into multivariable models—together with maternal characteristics, cervical-length measurements, and microbiologic tests—may improve risk stratification. Recent evidence demonstrates that combining first-trimester biomarkers such as pregnancy-associated plasma protein A (PAPP-A) and free β-human chorionic gonadotropin (β-hCG) with clinical variables improves prediction of preterm birth [13,20]. A recent study also confirmed associations between first-trimester biomarkers and subsequent risk of preterm birth and PPROM, reinforcing their potential utility in early pregnancy risk assessment [30].

Although emerging biomarkers such as matrix metalloproteinase-8 (MMP-8) show strong biological relevance due to their role in collagen degradation and neutrophil activation [29], most MMP-8 studies lacked eligible comparison groups or extractable data. Consequently, these biomarkers could not be included in the pooled analysis despite their pathophysiological importance [28].

### 4.3. Limitations

This review has several limitations. First, heterogeneity across studies was substantial, driven by differences in sampling time (admission, diagnosis, or pre-delivery), assay platforms (ELISA, chemiluminescent assays, multiplex systems), and non-standardized diagnostic criteria for PPROM and microbial invasion. Variability in population characteristics—including gestational age, maternal comorbidities, and regional microbiologic patterns—likely contributed further to between-study differences.

Second, most included studies were observational, limiting causal inference. Neonatal outcomes were often inconsistently reported or lacked extractable numerical data, preventing quantitative synthesis of clinically important endpoints such as microbial invasion of the amniotic cavity, histologic chorioamnionitis, and neonatal sepsis. Only four studies reported odds ratios or raw data suitable for potential pooling, which is below the threshold for valid meta-analysis. Thus, while IL-6 is associated with adverse outcomes, our conclusions reflect biomarker-level differences rather than outcome-based risk estimates.

Third, conference abstracts were excluded a priori, which may contribute to publication bias, although Egger’s test for serum IL-6 (the only analysis with ≥10 studies) did not suggest small-study effects (*p* = 0.21). Finally, all pooled estimates were based on summary statistics rather than individual participant data, which may limit precision.

### 4.4. Implications and Future Research

The present findings support IL-6—especially in amniotic fluid—as a clinically actionable biomarker for identifying intra-amniotic inflammation in PPROM and guiding personalized management. Its diagnostic accuracy surpasses commonly used markers such as CRP or leukocyte count, suggesting that IL-6 measurement may aid clinical decision-making regarding corticosteroid timing, antibiotic therapy, and delivery planning [16,18].

Future research should focus on multicenter prospective studies using standardized biomarker protocols and harmonized definitions of PPROM and intra-amniotic inflammation [23,30,31,32]. Additional biomarkers, including IL-1β, procalcitonin, MMP-8, and multiplex cytokine panels, warrant investigation using standardized quantitative methods. Integrating biomarker data with clinical scoring tools, ultrasound findings, and machine-learning prediction models may provide a more comprehensive and accurate approach to early identification of high-risk pregnancies [19,23,33].

Finally, linking first-trimester biochemical markers (PAPP-A, free β-hCG) with mid-gestational inflammatory biomarkers may enable development of multi-timepoint risk algorithms capable of predicting both PPROM and adverse neonatal outcomes across pregnancy [29,34].

## 5. Conclusions

This systematic review and meta-analysis demonstrate that maternal IL-6 concentrations, measured in either serum or amniotic fluid, are consistently and substantially elevated in pregnancies complicated by PPROM. Among all evaluated inflammatory biomarkers, IL-6 showed the strongest and most reliable association with PPROM, supporting its role as a central mediator of intra-amniotic inflammation and membrane weakening. Amniotic-fluid IL-6 showed the highest discriminatory performance, whereas serum IL-6 provided clinically meaningful, though comparatively less specific, information.

These findings highlight the potential clinical utility of IL-6 for early identification of women at increased risk of intra-amniotic infection, chorioamnionitis, and early preterm birth. Incorporating IL-6 into clinical evaluation—either alone or as part of a multimarker panel—may enhance risk stratification and guide timely interventions such as corticosteroid administration, antibiotic therapy, and individualized delivery planning.

However, meaningful heterogeneity across studies and the predominance of observational designs underscore the need for future research. Large, prospective, multicenter studies using harmonized biomarker thresholds, standardized sampling protocols, and comprehensive neonatal outcome reporting are essential for validating IL-6 as a diagnostic and prognostic tool. Studies integrating IL-6 with emerging biomarkers, imaging findings, or machine-learning-based prediction models may further improve diagnostic accuracy.

Overall, IL-6 appears to be a promising, clinically actionable biomarker for improving the early detection and management of PPROM, but further high-quality evidence is required before routine clinical implementation.

## Figures and Tables

**Table 1:** Example Search Strategies and Results.

Database	Search Strategy (Example)	Hits/Records Retrieved
PubMed/MEDLINE	(“preterm prelabor rupture of membranes” [Title/Abstract] OR “preterm premature rupture of membranes” [Title/Abstract] OR “PPROM” [Title/Abstract]) AND (“interleukin-6” [Title/Abstract] OR “IL-6” [Title/Abstract] OR “inflammatory marker” [Title/Abstract] OR “biomarker” [Title/Abstract]) AND (“maternal” [Title/Abstract] OR “serum” [Title/Abstract] OR “amniotic” [Title/Abstract] OR “plasma” [Title/Abstract])	238
Scopus	TITLE-ABS-KEY (“preterm prelabor rupture of membranes” OR “preterm premature rupture of membranes” OR “PPROM”) AND TITLE-ABS-KEY (“interleukin-6” OR “IL-6” OR “biomarker” OR “inflammatory marker”) AND TITLE-ABS-KEY (“maternal” OR “serum” OR “amniotic” OR “plasma”)	183
Web of Science (WoS)	TS = (“preterm prelabor rupture of membranes” OR “preterm premature rupture of membranes” OR “PPROM”) AND TS = (“interleukin-6” OR “IL-6” OR “inflammatory marker” OR “biomarker”) AND TS = (“maternal” OR “serum” OR “amniotic” OR “plasma”)	156

**Table 2:** Characteristics of included studies.

Study (Year)	Population (P)	Index Biomarker (I)	Comparator (C)	Outcomes (O)	Study Design	Sample Size (PPROM/ Control)
Romero 2015 [1]	PPROM	AF IL-6	Term controls	Inflammation	Case–control	180(90/90)
Yoon 2001 [2]	PPROM	Serum IL-6	Preterm labor w/o rupture	MIAC	Case–control	160(80/80)
Menon 2019 [3]	PPROM	Serum inflammatory markers	Term controls	Molecular inflammation	Case–control	96(48/48)
Musilová 2017 [4]	PPROM 24–34 wks	AF IL-6	Term intact membranes	MIAC, chorioamnonitis	Case–control	220(110/110)
Vink 2015 [6]	PPROM	Serum markers	Term controls	Cervical inflammation	Cohort	112
Kacerovský 2012 [7]	PPROM	AF IL-6	Term controls	Cytokine levels	Case–control	130(65/65)
Cobo 2012 [8]	PPROM	AF IL-6	Term controls	Adverse outcomes	Cohort	110(55/55)
Kacerovský 2009 [9]	PPROM 22–34 wks	AF IL-6	Term controls	MIAC	Cohort	185(PPROM only)
Cobo 2014 [11]	PPROM	AF cytokines incl. IL-6	Term + pre-term controls	Infection, neonatal outcomes	Case–control	95
Buhimschi 2020 [12]	PPROM	Serum + AF cytokines	Term controls	Inflammation pathways	Cohort	102(51/51)
Tsiartas 2013 [13]	PPROM	Serum IL-6, CRP	Term controls	IA infection	Cohort	210(105/105)
Keelan 2018 [14]	PPROM	Serum cytokines	Uncomplicated pregnancies	Cytokine profile	Case–control	88
Kim 2015 [15]	PPROM	AF IL-6	Term controls	Histologic chorioamni-onitis	Case–control	140(70/70)
Chaemsaithong 2016 [17]	PPROM	AF IL-6, AF biomarkers	Term controls	Intra-amniotic infection	Cohort	146(73/73)
Goldenberg 2008 [18]	PPROM	Serum biomarkers	Term pregnancies	Infection markers	Case–control	134(67/67)
Park 2017 [20]	PPROM	Serum IL-6	Healthy controls	MIAC prediction	Case–control	178 (89/89)
Lee 2012 [21]	PPROM	Serum cyto- kines	Term controls	Diagnostic accuracy	Case–control	120(60/60)
Paquette 2023 [22]	PPROM	IL-6 + biomarker panel	Term controls	Prediction modeling	Cohort	152(76/76)
Conde-Agudelo 2011 [23]	PPROM	Serum IL-6	Healthy controls	Preterm birth risk	Case–control	165
Madan 2023 [24]	PPROM	Serum CRP + IL-6	Term controls	Systemic inflammation	Case–control	118
Musilová 2015 [25]	PPROM	AF IL-6	Term labor	MIAC	Cohort	160(80/80)
Savasan 2010 [26]	PPROM	Serum IL-6	High-risk pregnancies	Predictive value	Cohort	143
Kacerovský 2022 [27]	PPROM	IL-6 + IL-8	Term intact membranes	Infection	Cohort	121

**Table 3:** Newcastle–Ottawa Scale (NOS) quality assessment of the included studies.

Study	Selection (0–4)	Comparability (0–2)	Outcome/Exposure (0–3)	Total (0–9)
Romero et al., 2015 [1]	4	1	3	8
Yoon et al., 2001 [2]	3	2	3	8
Menon et al., 2019 [3]	4	1	2	7
Musilová et al., 2017 [4]	4	1	3	8
Vink et al., 2015 [6]	**3**	**1**	**3**	7
Kacerovský et al., 2012 [7]	4	1	3	8
Cobo et al., 2012 [8]	3	1	3	7
Kacerovský et al., 2009 [9]	3	1	3	7
Cobo et al., 2014 [11]	3	1	3	7
Buhimschi et al., 2020 [12]	3	2	3	8
Tsiartas et al., 2013 [13]	3	1	3	7
Keelan et al., 2018 [14]	3	1	3	7
Kim et al., 2015 [15]	3	1	3	7
Chaemsaithong et al., 2016 [17]	3	2	3	8
Goldenberg et al., 2008 [18]	4	1	3	8
Park et al., 2017 [20]	3	2	3	8
Lee et al., 2012 [21]	3	1	3	7
Paquette et al., 2023 [22]	3	2	3	8
Conde-Agudelo et al., 2011 [23]	3	2	3	8
Madan et al., 2023 [24]	3	1	3	7
Musilová et al., 2015 [25]	3	1	3	7
Savasan et al., 2010 [26]	3	1	3	7
Kacerovský et al., 2022 [27]	**3**	**2**	**3**	8

**Table 4:** Summary of pooled meta-analysis results for maternal inflammatory biomarkers.

Biomarker	Specimen Type	No. of Studies	Pooled SMD (95% CI)	*p*-Value	*I*^2^ (%)
IL-6	Serum	15	1.72 (1.15–2.29)	<0.001	68
IL-6	Amniotic fluid	8	2.84 (2.01–3.67)	<0.001	79
CRP	Serum	10	0.98 (0.61–1.36)	<0.001	63
IL-8	Serum	5	0.45(0.12–1.03)	0.02	58
TNF-α	Serum	4	0.32(0.05–0.69)	0.04	50

## Data Availability

No new data were created or analyzed in this study.

## Supplementary Materials

The following supporting information can be downloaded at: https://www.mdpi.com/article/10.3390/medicina62010020/s1, PRISMA 2020 Checklist [35].

## Abbreviations

The following abbreviations are used in this manuscript:

AF	Amniotic fluid
CI	Confidence interval
CRP	C-reactive protein
ELISA	Enzyme-linked immunosorbent assay
Free β-hCG	free beta subunit of Human Chorionic Gonadotropin
IL-6	Interleukin-6
IL-8	Interleukin-8
MIAC	Microbial invasion of the amniotic cavity
NOS	Newcastle–Ottawa Scale
MMP-8	matrix metalloproteinase-8
PAPP-A	Pregnancy associated plasma protein-A
PPROM	Preterm prelabor rupture of membranes
REML	Restricted maximum likelihood
SMD	Standardized mean difference
TNF-α	Tumor necrosis factor-alpha
WoS	Web of Science
